# Association of HIVEP3 Gene and Lnc RNA with Femoral Neck Bone Mineral Content and Hip Geometry by Genome-Wide Association Analysis in Chinese People

**DOI:** 10.1155/2020/6929073

**Published:** 2020-10-13

**Authors:** Weiwei Hu, Jinwei He, Luyue Qi, Chun Wang, Hua Yue, Jiemei Gu, Hao Zhang, Yi Wang, Zhenlin Zhang

**Affiliations:** ^1^Shanghai Clinical Research Center of Bone Diseases, Department of Osteoporosis and Bone Diseases, Shanghai Jiaotong University Affiliated Sixth People's Hospital, Yishan Road 600, Shanghai 200233, China; ^2^Ministry of Education Key Laboratory of Contemporary Anthropology, Collaborative Innovation Center for Genetics and Development, School of Life Sciences, Shanghai, China; ^3^Human Phenome Institute, Fudan University, Shanghai, China

## Abstract

**Purpose:**

GWAS has successfully located and analyzed the pathogenic genes of osteoporosis. Genetic studies have found that heritability of BMD is 50%–85% while the other half is caused by hip geometric parameters and tissue horizontal characteristics. This study was designed to study the GWAS of osteoporosis in Shanghai Han population.

**Methods:**

We collected 1224 unrelated healthy young men (20–40 years old), young women (20–40 years old), and postmenopausal women (over 50 years old) who lived in Shanghai. BMD and hip geometric parameters were measured by dual-energy X-ray absorptiometry. The genomic DNA of peripheral blood was extracted and analyzed by using Illumina Human Asian Screening Array-24 + *v*1.0 (ASA) gene chip. Statistical analysis was carried out to evaluate the relationship between these SNPs and BMD and hip geometric parameters.

**Results:**

A total of 1155 subjects were included. We found that one SNP rs35282355 located in the human immunodeficiency virus type 1 enhancer-binding protein 3 gene (HIVEP3) and another 25 SNPs located in LINC RNA were significantly correlated with bone mineral content (BMC) in the femoral neck (*P*= 2.30 × 10^−9^, *P* < 5 × 10^−8^). We also found that the correlation between SNP rs35282355 and cross-sectional area (CSA) of hip geometry was a significant marginal statistical difference (*P* = 5.95 × 10^−8^).

**Conclusions:**

Through this study, we found that HIVEP3 gene and LINC RNA are potentially correlated with femoral neck BMC. These results provide important information for us to further understand the etiology and genetic pathogenesis of osteoporosis. In the future, we will expand the sample size to verify these loci and carry out molecular research.

## 1. Introduction

Osteoporotic fracture (OF) is the most severe clinical outcome of osteoporosis, which confers substantial morbidity, mortality, and social services expenditure in the elderly [1, 2]. At present, in the world, there have been more than 9 million fractures annually due to osteoporosis, of which 1.6 million were at the hip, 1.7 million were at the forearm, and 1.4 million were clinical vertebral fractures [1, 3]. In China, there was an obvious increase in the prevalence of osteoporosis from 14.94% before 2008 to 27.96% during the period spanning 2012–2015; this brings an enormous economic burden on the whole society [4]. Our study showed that the prevalence of vertebral fracture was 17.2% among Shanghai community-dwelling elders (over 60 years), 17.0% for males, and 17.3% for females. The prevalence among females increased with age [5]. The prevalence of osteoporosis in our country has increased over the past 12 years, affecting more than one-third of people aged 50 years and older [4].

Measurement of bone mineral density (BMD) has been used for the gold standard for the diagnosis of osteoporosis and the assessment of fracture risk. Variations in BMD can be explained by genetic effects, with which heritability ranges from 0.5 to 0.8 [6, 7]. To date, there are more than 100 candidate genes/loci associated with variations in BMD which have been identified in genome-wide association studies (GWASs) and their meta-analyses [8, 9]. Many countries performed GWAS and osteoporosis: in UK, they identified 203 single-nucleotide polymorphisms (SNPs) associated with BMD as estimated by quantitative ultrasound of the heel by GWAS in 142,487 individuals [10]. Icelandic deCODE Study (dCG) discovered multiple genetic loci associated with spine and femoral neck BMD [11]. The first multiethnic BMD GWAS used the Europeans as a discovery population, also the Chinese population and the African people in this study. This particular GWAS identified ADAMTS18 and TGFBR3 as bone mass candidate genes in different ethnic groups [9]. But in this study, there were only a Chinese hip fracture sample and 2955 Chinese BMD subjects for replication. We found that GWAS and BMD in China began in 2010, only a Chinese hip fracture sample (350 with hip OF and 350 healthy matched controls) [12]. After that, Tan et al. published GWAS and osteoporosis again and found ATP6V1G1 as a novel locus underlying osteoporosis and age at menarche in 2015 [13]. In Hong Kong, a GWAS about 800 unrelated Chinese women with extremely high or low BMD was performed and the JAG1 gene was identified as a candidate for BMD regulation in 2010 [14]. So far, the data of BMD and GWAS of Han nationality in China are very few. Through PubMed search, we found that most of the current studies on BMD and GWAS in China were based on these two data sets [12, 14].

However, BMD does not accurately describe either the strength or a specific geometric configuration of bone tissue [15]. Our previous study also proved that hip geometry parameters are risk factors for hip fracture and that these are independent of BMD measurements [16]. Up to now, there were a few studies which performed GWAS on hip geometry measures [17]. By GWAS, femoral neck-shaft angle (NSA) was found for SNPs located on chromosome 2q11.2 (TBC1D8) in EU populations [17]. Another GWAS study in 1000 Caucasians found that rs7430431, in the receptor transporting protein 3 (RTP3) gene, was identified in strong association with hip geometry, the buckling ratio (BR), and femoral cortical thickness (CT), an index of bone structural instability [18]. By referring to the PubMed literature, there were only few papers on GWAS and hip geometry in the world, and there was no large sample about GWAS and hip geometry about Chinese Han people.

To identify genetic variants that influence BMD in different ethnic groups, we performed a GWAS on 1224 unrelated Chinese women and men in Shanghai with BMD parameters. In the present study, we also aimed to report the GWAS of hip geometry of these subjects, which is thought to be related to the risk of hip fracture, identified BMD-associated genes. Our study also was used as input information for subsequent gene enrichment analysis to assess their potential biological roles in osteoporosis pathophysiology.

## 2. Materials and Methods

### 2.1. Subjects

In total, 1224 unrelated subjects were collected, including 366 young men aged 20–40 years, 239 young premenopausal women aged 20–40 years, and 619 postmenopausal women aged 50–80 years. All subjects were Chinese Han people selected for inclusion in the study. The inclusion criteria were as follows: healthy men and women according to the following excluding criteria. Exclusion criteria in accordance with our previous articles were used to exclude individuals from the study [5, 16, 19, 20]: (1) serious effects from cerebrovascular disease; (2) other diseases, such as diabetes mellitus, chronic renal disease, chronic liver disease, rheumatoid arthritis or collagen disease, hyperthyroidism, and recent major gastrointestinal disease; (3) evidence of other metabolic or inherited bone diseases; (4) significant diseases of any endocrine organ that would affect bone mass; (5) any neurological or musculoskeletal condition; and (6) any form of calcium and vitamin-D therapy in three months or taking antiosteoporotic drugs (e.g., bisphosphonates, selective estrogen receptor modulators, and calcitonin). Postmenopausal women who had experienced early menopause (before 45 years of age) were excluded. All study subjects belonged to the Chinese Han ethnic group. All were residents of Shanghai City, located in the mideastern coast of China. The study was approved by the ethics committee of the Shanghai Jiao Tong University Affiliated Sixth People's Hospital.

### 2.2. BMD and Hip Geometry Parameters Measurements

The BMD of the lumbar spine 1–4 (L1-4), the left proximal femur, bone mineral content (BMC), and hip geometry parameters were measured by DXA on a GE-LUNAR Prodigy (Lunar Corp., Madison, WI). All hip scans were reprocessed using enCore Installation 13.10.078 software (GE Healthcare). As our previous study described, this software includes advanced hip analysis, which provides the following structural parameters: hip axis length (HAL), cross-sectional moment of inertia (CSMI), cross-sectional area (CSA), and femoral strength index (SI) [20]. The root mean square coefficient of variability (RMSCV%) values of BMD and hip geometry were obtained from our previous studies. The RMSCV% values of the DXA measurements at L1-4, the femoral neck, trochanter, and total hip were 1.39%, 2.22%, 1.41%, and 0.70%, respectively [16]. The RMSCV% values of the HAL, CSMI, CSA, and SI were 1.09%, 4.91%, 2.49%, and 6.07%, respectively [16, 19, 20].

### 2.3. Genotyping

High-throughput genome-wide SNP genotyping was performed the Illumina Infinium Asian Screening Array-24 + *v*1.0 BeadChip technology containing 846000 SNPs and following the manufacturer's protocol.

### 2.4. Statistical Analysis

The data are given as the mean ± SD for normal data. We divided the data into three groups according to age, and differences between groups were assessed using an ANOVA test with Fisher's protected least significant difference post hoc test for normal data. The threshold for the replication significance was set at *P* < 0.05. Statistical analyses were performed using SPSS version 21.0 for Windows (SPSS Inc.).

### 2.5. Phenotype Normalization

Phenotypes were firstly rank-transformed in order to attenuate the impact of outliers and nonnormal distributions. A random forest regression is performed using each phenotype rank as a target while using sex and age as predictors. The out-of-bag estimates (age-sex expected phenotype ranks) are subtracted from the phenotype ranks. Again, this age-sex adjusted rank is rank-transformed as the final phenotype rank and used for later analysis.

### 2.6. Genotype Imputation

Genotype imputation is performed by a commercial imputation engine named GenoImpute. We obtain a mean sample-level *r*^2^ of 0.736 estimated by 1% holdout SNPs on the array. Different from other off-the-shelf imputation engines, this engine produces a continuous allele dosage as well as three-genotype probability distribution which reflects the reality of genotype uncertainty. The allele dosage is used for later analysis.

### 2.7. Phenotype Rank: SNP Association Test

We employ the Spearman correlation test with a permutation-based exact *P* value to test phenotype rank–SNP associations. Up to 10^10 simulation is performed. According to the statistical analysis of the data mapping analysis: the whole genome trend chi-square test results in SNPs, chromosome, physical location, and *P* value of the input *R* software to generate a genome-wide association analysis of the Manhattan map and Quantile-Quantile plots map (Q-Q map).

## 3. Results

### 3.1. Basic Characteristics of the Study Subjects

**Figure 1:**
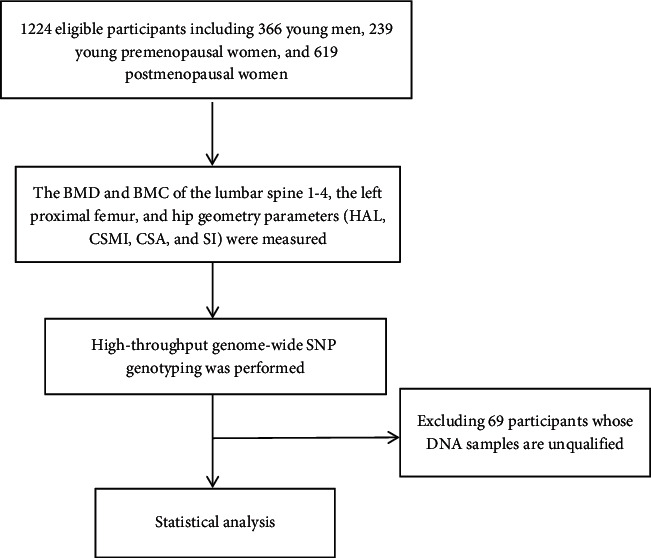
Flow diagram of the study. This is a brief flow chart of this experiment, indicating the number of screening, screening steps, and other details.

**Figure 2:**
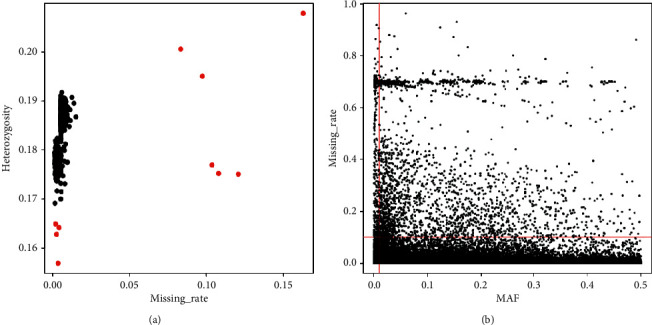
The principal analysis of the Han population samples in Shanghai. There were 570378 SNPs on the chip. SNPs were filtered with MAF < 0.01 or p-HWE < 0.01 or miss rate > 0.1 and retained 474773 SNPs. (a) Sample QC. (b) Marker QC.

Figure 1 shows the flow diagram of this study. We obtain 1167 raw chip samples. By filtering samples with low heterozygosity (<0.165 according to visual outlier inspection) and a high missing rate (>0.05), we retained 1155 samples. There are 570378 SNPs on the chip. We filtered SNPs with MAF < 0.01 or p-HWE < 0.01 or miss rate > 0.1 and retained 474773 SNPs (Figure 2).

The basic characteristics of the samples are summarized in Table 1. BMD of the lumbar spine, femoral neck, and total hip in postmenopausal women were 0.980 ± 0.152 g/cm^2^, 0.784 ± 0.126 g/cm^2,^ and 0.846 ± 0.131 g/cm^2^, respectively. Hip and spine BMD or BMC were lower in postmenopausal women than in young men and premenopausal young women. This significant difference observed here is consistent with the previous findings. In the young male group, the hip geometric parameters HAL, CSMI, CSA, and SI were 110.82 ± 6.40 mm, 14034 ± 3693 mm^4^, 164.49 ± 26.29 mm^2^, and 1.59 ± 0.13, respectively. In the young men group, there were higher HAL, CSMI, and CSA than the other two groups.

### 3.2. Relationship between BMC and GWAS, Hip Geometric Parameters, and GWAS

**Figure 3:**
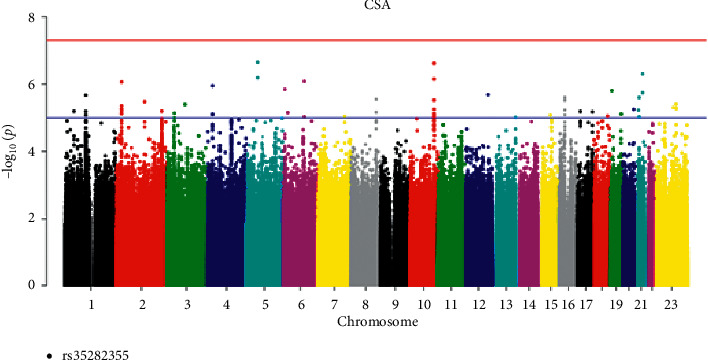
Manhattan plot of CSA (chromosomal plot).

Our quantitative trait analyses identified SNPs by a genome-wide significant threshold (*P* < 5 × 10^−8^).  Table 2 shows the distribution of *P* values of GWASs across the genome in our Chinese samples. We found some significant SNPs with *P* < 5 × 10^−8^. We listed some SNPs for CSA in Table 2. There is a marginal difference between SNP rs35282355 (*P* = 5.95 × 10^−8^) located in human immunodeficiency virus type 1 enhancer-binding protein 3 (HIVEP3) gene and CSA, suggesting that it is a susceptible site of CSA (Figure 3).

**Figure 4:**
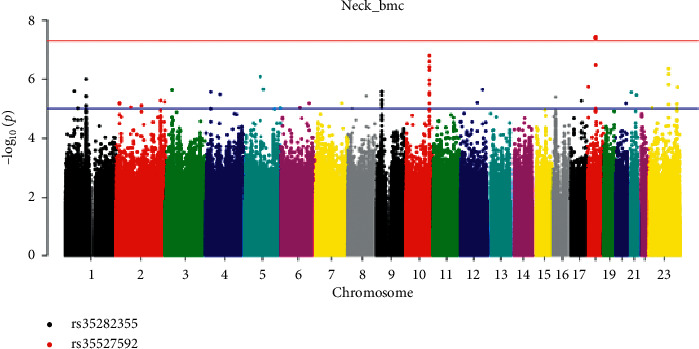
Manhattan plot of neck BMC (chromosomal plot).

In the femoral neck BMC phenotype, 26 SNPs (see Table 3) reached the genome-wide significance level (*P* < 5 × 10^−8^) (Table 3). Interestingly, the strongest association was found at SNP rs35282355 located in the intron of the HIVEP3 gene, with *P* = 2.30 × 10^−9^ for neck BMC (Figure 4), which has not been implicated in GWAS of BMD previously. This locus was most strongly associated with neck BMC. While the other 25 SNPs with significant statistical significance were located in the long intergenic nonprotein coding RNA (LINC RNA).

## 4. Discussion

In this study, we performed a GWAS of lumbar spine BMD, proximal femur BMD, lumbar spine BMC, proximal femur BMC, and hip geometry parameters in 1155 participants from Shanghai. The GWAS study of osteoporosis was carried out for the first time in the Han population in Shanghai. We found a SNP rs352355 (*P* = 5.95 × 10^−8^) of HIVEP3 gene located on 1p34.2 was significantly associated with CSA, suggesting that it was the susceptible site of CSA, a geometric parameter of the hip. At the same time, this locus was found to have genome-wide significance with femoral neck BMC (*P* = 2.30 × 10^−9^). We identified rs35282355 in HIVEP3 gene that was associated with both CSA and femur neck BMC.

HIVEP3, also known as Schnurri-3 (SHN3), was a zinc finger protein and an important regulator of bone formation. This gene had 17 exons, of which rs35282355 was located in the intron region. SHN3-deficient mice exhibited osteosclerosis due to increased osteoblast activity and increased bone mass [21]. In osteoblasts, SHN3 was a component of the trimer complex between Runx2 and E3 ubiquitin ligase WWP1. Because WWP1 promoted ubiquitination of Runx2 and protease-dependent degradation, the complex inhibited Runx2 function and gene expression involved in extracellular matrix mineralization. SHN3 controls protein levels by degrading Runx2 which was an essential regulator of osteoblastogenesis, the factor that had been validated [21–23]. In 2013, Shim et al. [24] found that SHN3 is an attenuator of extracellular regulated protein kinases (ERK) activity. In osteoblasts, ERK was the key to signal transduction from surface receptors to nuclei. ERK activity played a role downstream of WNT signal transduction. *D* domain in SHN3 mediated the interaction and inhibition of ERK activity and osteoblast differentiation [24]. The knock-in of SHN3 gene mutation eliminated this interaction, which led to abnormal ERK activation and excessive activity of osteoblasts in vivo. In addition, the study also showed that hybridization with Lrp5^−/−^ mice partially saved the osteosclerosis phenotype of SHN3^−/−^ mice, which corresponded to the ability of SHN3 to inhibit ERK-mediated GSK3 beta inhibition. Induced knockout of Shn3 in adult mice led to high bone mass phenotypes, providing evidence that temporary blockade of these pathways had the potential for the treatment of osteoporosis in adult mice [24]. By searching PubMed, this study was the first to find that the HIVEP3/SHN3 gene had a potential relationship with hip bone mass in the Chinese Han population of GWAS and reached the level of genome-wide significance. However, at present, no potential correlation between this gene and hip bone mass had been found in other GWAS and bone mineral density studies. In our future experiments, we can further expand the sample size to verify and study the function of the gene.

The latest article on the meta-analysis of GWAS of hip geometry phenotypes provided a defined set of genes related to biological pathways relevant to hip geometry [25]. The results found that several hip geometry signals overlapped with BMD. SNPs of protein phosphatase 6 regulatory subunit 3 (PPP6R3), fibroblast growth factor receptor 4 (FGFR4), and PDZ and LIM domain 7 (PDLIM7) genes, which had been confirmed to play an important role in osteogenesis, were found associated with hip geometry. Therefore, we speculate that although rs35282355 in HIVEP3 gene is an intron, alteration of HIVEP3 expression by Runx2 is involved in osteogenesis to affect bone geometry. But small sample size in our study may limit the statistical power to detect the association between HIVEP3 gene and hip geometry.

Further, we also observed significant associations with 25 SNPs and BMC of femoral neck reached the genome-wide significance level, all of which were located at long intergenic nonprotein coding RNA 907 (LINC00907) on 18q12.3. LINC00907 belonged to long nonprotein coding RNA (LncRNA); these results suggested that LncRNA was a susceptible site for bone mass of the femoral neck. The transcription length of LncRNA ranged from 200 nt to 10 kb and was widely present in the nucleus and cytoplasm of cells [26]. Because LncRNA lacked reading frames, they almost never participated in protein-coding. More evidence showed that LncRNA transcribed by DNA was not useless; they participated in a variety of biological processes and regulated gene expression at the RNA level [26, 27]. About 15779 LncRNAs had been recorded in the human genome (https://www.gencodegenes.org/) [28]. At present, 51 kinds of LncRNA had been identified as differentially expressed in postmenopausal osteoporosis patients compared with the healthy control group and participated in the pathogenesis of the disease [28, 29].

We looked up the upstream and downstream locations of LINC00907 and found that the downstream Rit2 gene was associated with Parkinson's disease, schizophrenia, and autism. Rit2 protein products are members of Ras superfamily and play an important role in many important cellular functions, such as differentiation and survival. We predict that LINC00907 may regulate the proliferation and differentiation of osteoblasts or osteoclasts by regulating the Rit2 gene. At the upstream of LINC00907, phosphatidylinositol 3 kinase catalytic subunit 3 (PIK3C3), an 887 amino acid lipid kinase, was found to regulate intracellular membrane transport and autophagy. We speculate that LINC00907 may also regulate apoptosis of osteoblasts and osteoclasts by regulating the PIK3C3 gene. A series of studies had proved that many kinds of LncRNAs participated in the regulation of osteoblast proliferation and function through competitive endogenous RNA to transcript, posttranscript, and epigenetic level [28, 30, 31]. Jin et al. also found that 70 lncRNAs were significantly expressed in peripheral blood lymphocytes of postmenopausal osteoporosis patients compared with the control group [32]. Wang et al. found that LINC00311 was highly expressed in the osteoporotic rat model. They found that LINC000311 induced osteoclast proliferation and inhibited osteoclast apoptosis through the Notch pathway [33]. Recently, Zeng et al. [34] identified two LncRNAs polymorphisms at 5q14.3, which mapped in MEF2C antisense RNA 1 (MEF2C-AS1) region. It has been confirmed that the gene is related to bone mass [35]. However, further studies were needed to elucidate the role of abnormal LncRNA expression in the pathogenesis of osteoporosis, as well as its pathological and molecular mechanisms. Therefore, the LINC00907 found in this study is significantly correlated with femoral neck bone mass, and these polymorphisms provide a basis for the follow-up function study in the pathophysiology of osteoporosis.

Our study provides some clues for the basic research of osteoporosis genetics and future biological experiments. Our study also has some shortcomings. Our research has not identified all the genes related to BMD found by predecessors but found some new genes related to BMD. This may be due to the fact that only a part of the lost heritability can be found in our study. As a supplement to GWAS and gene expression differences, more experimental studies are needed to verify our findings in the future. Because the various research design methods and genetic statistics methods of GWAS research cannot fundamentally eliminate the false positives caused by population mixing and multiple comparisons, we need to ensure the true association between genetic markers and diseases through repeated studies. Our findings suggested potentially new bone regulatory pathways that warrant further study. Subsequently, we will expand the research subjects to verify the experimental results.

## Figures and Tables

**Table 1:** Basic characteristics of the studied samples.

Traits	Total sample	Young men	Young women	Postmenopausal women
N	1155	341	220	594
Age (y)	47.88 ± 17.85	28.86 ± 6.12^*∗*^	32.47 ± 5.87^*∗*^	64.28 ± 7.10^*∗*^
Height (m)	162.0 ± 9.7	173.0 ± 6.0^*∗*^	160.1 ± 5.3^*∗*^	154.8 ± 5.1^*∗*^
Weight (kg)	62.05 ± 11.06	70.62 ± 10.20^*∗*^	55.93 ± 8.44^*∗*^	58.67 ± 8.76^*∗*^
Spine BMD (g/cm^2^)	1.075 ± 0.166	1.139 ± 0.139^*∗*^	1.172 ± 0.121^*∗*^	0.980 ± 0.152^*∗*^
Neck BMD (g/cm^2^)	0.887 ± 0.161	1.003 ± 0.139^*∗*^	0.924 ± 0.112^*∗*^	0.784 ± 0.126^*∗*^
Total hip BMD (g/cm^2^)	0.927 ± 0.150	1.015 ± 0.136^*∗*^	0.959 ± 0.117^*∗*^	0.846 ± 0.131^*∗*^
Spine BMC (g)	58.58 ± 13.73	69.13 ± 11.85^*∗*^	60.96 ± 9.06^*∗*^	49.72 ± 10.24^*∗*^
Neck BMC (g)	4.36 ± 1.03	5.35 ± 0.84^*∗*^	4.36 ± 0.61^*∗*^	3.65 ± 0.62^*∗*^
Total hip BMC (g)	28.17 ± 5.87	33.49 ± 4.9^*∗*^	27.07 ± 3.78^*∗*^	24.79 ± 4.19^*∗*^
HAL (mm)	102.32 ± 8.49	110.82 ± 6.40^*∗*^	98.25 ± 5.69	98.17 ± 6.08
CSMI (mm^4^)	9215 ± 4192	14034 ± 3693^*∗*^	7865 ± 2677^*∗*^	6953 ± 2138^*∗*^
CSA (mm^2^)	139.47 ± 236.98	164.49 ± 26.29^*∗*^	132.79 ± 18.97	127.61 ± 340.99
SI (dimensionless)	1.65 ± 4.23	1.59 ± 0.13	1.68 ± 0.48	1.67 ± 6.09

Continuous variables expressed as mean ± SD. ^*∗*^Statistical differences between groups, *P* < 0.05.

**Table 2:** The results of CSA associated SNPs in GWAS of the Han population in Shanghai (partially).

Phenotype	SNP	Alleles	*P*	Gene
CSA	rs35282355	G : A	5.95*E* − 08	HIVEP3
CSA	rs7071262	T : G	7.05*E* − 07	Null
CSA	rs7907051	G : A	2.42*E* − 07	Null
CSA	rs187172215	T : C	8.64*E* − 07	KLHL29
CSA	rs12152026	T : C	4.99*E* − 07	Null
CSA	rs117963651	G : A	6.48*E* − 07	Null
CSA	rs190389312	A : G	2.26*E* − 07	Null
CSA	rs17592374	T : C	8.03*E* − 06	Null
CSA	rs61791524	A : G	7.84*E* − 06	Null
CSA	rs61791526	T : G	7.84*E* − 06	Null
CSA	rs138917572	C : T	1.44*E* − 06	Null
CSA	rs647297	G : A	6.99*E* − 06	Null
CSA	rs200674613	T : C	8.30*E* − 07	Null
CSA	rs146148517	C : G	9.25*E* − 06	Null
CSA	rs117331232	C : T	9.02*E* − 06	Null
CSA	rs7006122	T : G	6.81*E* − 06	Null
CSA	rs5969489	G : A	4.95*E* − 06	Null
CSA	rs200674613	T : C	8.30*E* − 07	Null

**Table 3:** The results of femoral neck BMC associated SNPs in GWAS of the Han population in Shanghai (partially).

Phenotype	SNP	Alleles	*P*	Gene
Neck_bmc	rs35282355	G : A	2.30*E* − 09	HIVEP3
Neck_ bmc	rs35052221	A : G	3.66*E* − 08	LINC00907
Neck_ bmc	rs35250842	T : C	3.66*E* − 08	LINC00907
Neck_ bmc	rs35073620	C : T	3.66*E* − 08	LINC00907
Neck_ bmc	rs7238496	C : G	3.66*E* − 08	LINC00907
Neck_ bmc	rs62082201	C : T	3.66*E* − 08	LINC00907
Neck_ bmc	rs1461721	A : G	4.07*E* − 08	LINC00907
Neck_ bmc	rs35959504	A : G	3.66*E* − 08	LINC00907
Neck_ bmc	rs2219638	A : T	4.07*E* − 08	LINC00907
Neck_ bmc	rs1381349	C : T	4.07*E* − 08	LINC00907
Neck_ bmc	rs9304268	A : T	4.07*E* − 08	LINC00907
Neck_ bmc	rs7234053	A : C	4.07*E* − 08	LINC00907
Neck_ bmc	rs35527592	T : A	3.33*E* − 07	LINC00907
Neck_ bmc	rs1461719	T : C	4.07*E* − 08	LINC00907
Neck_ bmc	rs10502780	C : A	4.07*E* − 08	LINC00907
Neck_ bmc	rs7236024	G : A	4.07*E* − 08	LINC00907
Neck_ bmc	rs7235905	C : T	4.07*E* − 08	LINC00907
Neck_ bmc	rs12962756	A : G	4.07*E* − 08	LINC00907
Neck_ bmc	rs12457855	C : A	4.07*E* − 08	LINC00907
Neck_ bmc	rs34703522	G : A	4.07*E* − 08	LINC00907
Neck_ bmc	rs12457879	C : T	4.07*E* − 08	LINC00907
Neck_ bmc	rs12457881	C : G	4.07*E* − 08	LINC00907
Neck_ bmc	rs7241462	A : G	4.07*E* − 08	LINC00907
Neck_ bmc	rs12967873	A : G	3.66*E* − 08	LINC00907
Neck_ bmc	rs4890391	A : G	4.07*E* − 08	LINC00907
Neck_ bmc	rs1461718	A : G	4.07*E* − 08	LINC00907

## Data Availability

The data used to support the findings of this study have not been made available because of restrictions by the ethics in the hospital in order to protect patients' privacy.
